# The Nature and Method of Medical Societies1Presidential address, read at the Thirty-sixth Annual Meeting of the Medical Association of Central New York, at Buffalo, October. 1905

**Published:** 1906-02

**Authors:** Charles G. Stockton

**Affiliations:** Buffalo, N. Y.; Professor of Medicine in the University of Buffalo


### BUFFALO MEDICAL JOURNAL.

VOL. LXI. FEBRUARY, 1905. No. 7

### ORIGINAL COMMUNICATIONS.

### The Nature and Method of Medical Societies.[1]

BY CHARLES G. STOCKTON, M. D., Buffalo, N. Y.
Professor of Medicine in the University of Buffalo.

IN one of his cogent and illuminating remarks, Schopenhauer, in his essay "On Books and Reading," says: "When we read, another person thinks for us; we merely repeat his mental process. In reading, the mind is in fact only the playground of another's thoughts." Again he remarks that, many learned persons have read themselves stupid, * * * and, the spring never free from the pressure of some foreign body, at last loses its elasticity; and so does the mind if other people's thoughts are constantly forced upon it." While this is seemingly a contradiction to Bacon's saying that, "Reading maketh a full man," it may be found upon maturer reflection that there is truth in both of these views expressed. If it is true, as Schopenhauer contends, that when we read, another person thinks for us, then it would seem to follow that he who writes a book is one at least who thinks. Now, however this may be, the whole question of writing and reading papers is one of great moment to physicians, and especially is this true when they meet in medical societies for the expression of their professional thought. These organisations doubtless had their inception for the purpose of discussing subjects in which all were mutually interested, in comparing observations and reflections connected with the practical and theoretical sides of medicine.

An important feature in the early medical societies seems to have been a dissertation by some learned individual, followed by discussion, much as we carry on matters today. In fact it would be difficult to secure interest in the proceedings of a medical society unless there was arranged a formal programme of papers, and it would be a hard task for the members of the medical profession to act in touch with each other unless the papers were pub-

1. Presidential Address, read at the Thirty-sixth Annual Meeting of the Medical Association of Central New York, at Buffalo, October. 1905

lished and read as well as written. In early days when few books and comparitively few papers were prepared and published, the appearance of a new article was a matter of greater importance than now; it excited more thought and more discussion, and even in the absence of modern methods of filing and reference, these old contributions were doubtless longer preserved and better remembered. In our time we find that the number of men competent to write papers is without number, and the interests in medicine are so varied and so changing that there naturally results such a mass of contributions that it taxes the best of us to keep abreast of even one department of medical literature. It strains the ingenuity of the programme committees to arrange the work so that a proper representation of medical thought, from various sections of the country, can find expression in the short time which we are able to devote to any single meeting. We find ourselves in the dilemma of asking for more papers than we can comfortably listen to and appropriate, or than we can read and digest when they are finally published in the journals. These remarks bring up familiar subjects of discussion at every meeting of the programme committee of all large medical societies. The question is not only a persistent one, but it is important. It may perhaps best be discussed under two heads:

First, What course, if followed, would prove most beneficial to the individuals of the profession?

Second, What would be best for medical science as a whole?

As to the individual physician, I have no doubt that it is of the greatest importance for him to write papers. For, usually, when one writes a paper he does his best thinking; he recalls his best experience, and he often records important facts. He becomes better acquainted with his own knowledge and efficiency, and his familiarity with these is usually somewhat sharpened as the result of the discussion which he invites. As to those who listen to the reading of papers, doubtless some derive advantage and others disadvantage according to their state of mind. To one who thinks for himself, who squares his reflections to the line of medical principles, who has systematically trained his mind to just and searching criticisms, and who is able to formulate his ideas so that he may make conclusions, to such a listener, undoubtedly great benefits arrive. But to the man who merely listens to the reading of papers without having roused within him the critical spirit and a strong desire to have the matter rightly concluded, who hopes that by hearing others read papers he may absorb information without laboring for it; who, in short, attends a medical meeting for the advantage of what he may pick up, surely, to such an individual the hearing read of a large number of papers must be a rather poor way of disposing of his time.

It would seem that the greatest advantage to an individual would come from the writing of papers and the discussions. In an ordinary programme it is impossible for each man to write and each man to discuss every paper. By what course can the remaining members of the society obtain the greatest good from the meeting? Undoubtedly he can achive the most by listening to papers with the mental attitude of one who proposes to discuss them; also by making memoranda of those statements which he is prepared neither to accept unconditionally nor to discard as altogether erroneous. Suggestive statements, yet of a kind that hardly carries conviction, nevertheless make us think and are often more profitable than those in which we readily concur. If we would only bring with us to a medical meeting the mind enriched by thinking carefully upon our own observations and a desire to criticise with the single end of advancing medical thought to a higher plane and eliminating misconception and error, then attendance at a medical meeting would be as valuable to the man who keeps his seat as to him who writes the articles or discusses them. In a word, if we can secure the right spirit in the units which compose a medical organization, we may take the greatest satisfaction in the transactions of an aggregation of units making up the society. It appears to me that the most useful society is not necessarily that one before which is read the most valuable papers; not necessarily that one at whose meeting the discussion is most general. As a matter of course, the papers should be of the best and the discussion should be wide, but neither of these necessarily reaches the majority of the members present, except those non-participating members who keep their thoughtful and critical minds in a proper attitude towards the proceedings. With such a spirit as this pervading a medical society, its work cannot fail to be admirable and profitable to each and all concerned.

Now, as to the second part of the question—What conduct in the society is best for medical science as a whole? Is it that there shall be many papers and a large publication, or few papers and much discussion, or systematic outlining of the work, as in formal symposia, or what? All these methods and many others are more or less in active operation in the innumerable medical societies of the country. The transactions are voluminous; one cannot remember even the titles of the new books; and the articles contributed to medical journals are innumerable. "Herodotus writes that Xerxes wept at the sight of his army which stretched further than the eye could reach, in the thought that of all these, after a hundred years, not one would be alive. And in looking over a huge catalogue of new books one might weep at thinking that, when ten years have passed, not one of them will be heard of." (Schopenhauer). Saddening as this reflection may be, it cannot mean that the writing of these books is useless. By the same argument, we might reason that individual lives are useless. Just as by the succession of lives the race is preserved and development insured, so by the procession of medical books do we keep alive medical interest, stimulate medical thought, and occasionally rise to better planes.

Those programmes seem to grow in favor which provide a limited number of papers and arrange beforehand for adequate discussion of these contributions. Undoubtedly this curtails the number of valuable contributions that should be put on record, and therefore the plan is not suited to all societies alike. Certain bodies like that of the "International Congress for the study of tuberculosis," held during the present month in Paris, is a society especially suited to the recording of contributions on a single subject from all parts of the world. At such a meeting discussions must necessarily be limited; it is of importance to gather together contributions. At the other extreme are those clinical societies, often connected with hospitals, in which personal observations are reported and the widest and freest discussion possible, entertained.

In organisations like the Medical Association of Central New York, it would seem the wisest plan to follow the middle course; otherwise, to invite a full number of contributions, spontaneous in character, avoiding too many formal symposia, urging the number of brief, spirited discussions and expecting each individual that mental attitude toward the meeting which of itself will secure a profitable assembly and can hardly escape making it brilliant and one to be remembered. It is my hope that this meeting will prove to be of this character.

436 FRANKLIN STREET.

